# Ultrasonographic features of cervical lymph node metastases from medullary thyroid cancer: a retrospective study

**DOI:** 10.1186/s12880-022-00882-7

**Published:** 2022-08-29

**Authors:** Xiaofeng Ni, Shangyan Xu, Weiwei Zhan, Wei Zhou

**Affiliations:** 1 Department of Ultrasound, Ruijin Hospital, Shanghai Jiao Tong University School of Medicine, 197 Ruijin Er Rd, Shanghai, 200025 China; 2 Department of Ultrasound, RuiJin Hospital Lu Wan Branch, Shanghai Jiao Tong University School of Medicine, Shanghai, China

**Keywords:** Medullary thyroid cancer, Papillary thyroid cancer, Lymph node, Metastases, Ultrasound

## Abstract

**Background:**

To investigate sonographic features of cervical lymph node metastases from medullary thyroid cancer (LNM-MTC), as compared with lymph node metastases from papillary thyroid cancer (LNM-PTC).

**Methods:**

A total of 42 MTC patients with 52 metastatic LNs and 222 PTC patients with 234 metastatic LNs who were confirmed by fine needle aspiration and post-operative pathology, were enrolled in this study. The clinical characteristics and sonographic features of LNs were compared between the two groups. Univariate analysis and multivariate logistic regression analysis were performed on the sonographic features of LNs, including short and long-axis diameter, long-axis diameter/short-axis, shape, border, hilum, echogenicity, calcifications, cystic change and vascularity pattern. The discriminating performance was assessed with the area under the receiver operating characteristic curve (AUC).

**Results:**

The mean age of patients with LNM-MTC was older than that of patients with LNM-PTC (46.81 ± 13.05 vs 39.09 ± 12.05, *P* < 0.001). No differences were observed in gender, location, long-axis diameter/short-axis, shape, border, echogenicity, cystic change and vascularity pattern between LNM-MTC and LNM-PTC groups (*P* > 0.05, for all). However, long-axis and short-axis diameter, hilum and calcifications were statistically different between these two groups (*P* < 0.05, for all). The AUC of discriminate value between LNM-MTC and LNM-PTC was 0.808 (95% confidence interval 0.739–0.877).

**Conclusion:**

Compared with LNM-PTC, LNM-MTC tended to have the sonographic characteristics of larger size, absence of hilum, and less calcifications, and awareness of these features might be helpful to in the diagnosis of LNM-MTC.

## Background

As a neuroendocrine tumor, medullary thyroid carcinoma (MTC) is originated from parafollicular cells (C cells), which could secrete calcitonin [1]. MTC accounts for 5–8% of all thyroid malignant nodules [2], and it is prone to present lymph node metastasis (LNM), with a high recurrence rate and poor prognosis. Even after the initial surgery, the recurrence rate is still up to 40–66% [3]. Therefore, early diagnosis of regional lymph nodes is important for determining surgical strategy, which may be helpful to improve quality of life and reduce the mortality rate.

Ultrasound(US) is an effective method to distinguish benign from metastatic lymph nodes(LN), with a high specificity, however, the sensitivity is low for LNMs, especially for central LNs [4, 5]. Previous studies showed that specific sonographic features of metastatic LNs from thyroid carcinoma included microcalcifications, hyperechogenicity, partially cystic change, peripheral or diffusely increased vascularization [6, 7]. However, the results of these studies were mainly based on lymph node metastases from papillary thyroid cancer(LNM-PTC), which might not be applicable to lymph node metastases from medullary thyroid cancer(LNM-MTC). To the best of our knowledge, only a few studies with limited cases had mentioned the sonographic features of LNM-MTC. The meta-analysis of US of metastatic lymph nodes in thyroid cancer only involved one case of MTC [8]. Therefore, this study aimed to investigate the clinical and sonographic characteristics of LNM-MTC compared with LNM-PTC.

## Methods

### Patients

This retrospective study was approved by Ethics Committee of the Ruijin Hospital, Shanghai Jiao Tong University School of Medicine, with waiver of informed consent. Consecutive patients with suspicious LNs who underwent US and fine needle aspiration (FNA) at our hospital from January 2014 to December 2020 were reviewed. Finally, 42 patients with 52 LNM-MTC and 222 patients with 234 LNM-PTC, who were confirmed by preoperative FNA and postoperative pathology, were enrolled in our study.

The inclusion criteria were as follows: (1) patients with suspicious cervical LNs which were evaluated by US examination and FNA; (2) US was performed by senior physicians with more than 10 years of experience in ultrasound operation; (3) LN dissection was performed, and LN was confirmed with post-operative pathology. Exclusion criteria were as follows: (1) patients without complete medical and US image information; (2) patients with history of neck irradiation; (3) cytological results were undiagnosable or unsatisfactory; (4) patients associated with other LN diseases, such as lymphoma, Kikuchi-Fujimoto disease, LN tuberculosis or metastasis from other diseases.

### Ultrasonography examination and FNA

During the sonographic examination, the patient lay on a bed in the supine position, and the anterior area of the neck was fully exposed. US examinations were performed with a 4- to 13-MHz linear probe (Mindray Resona7, China; MyLab 90, EsaoteSpA, Genoa, Italy & iU22, Philips, Seattle,WA, USA) by 1 of 3 radiologists with more than 10 years of experience in thyroid and LN sonography (XFN, SYX, WZ). The gray scale and Doppler US were carefully adjusted to obtain the best image. Images of each suspicious LN were obtained in both transverse and longitudinal orientations, and images were recorded and uploaded for later retrospective analysis.

The sonographic appearances of LNs were assessed carefully, including location, long-axis diameter (L), short-axis diameter(S), Long-axis diameter/short-axis (L/S), shape, border, cortex echogenicity, hilum, calcifications, cystic change and vascularity pattern. Location was divided lateral region (level I–V) and central region (level VI or VII). If multiple LNMs were found in the same region, only the largest one was selected. Long-axis and short-axis diameters were obtained at the maximum of the longitudinal section of LN. L/S was classified into ≤ 2 and > 2. Shape was assessed as irregular/regular. Border was divided into sharp and unsharp. The cortex echogenicity was determined as hyperechoic, isoechoic, and hypoechoic, as compared with the surrounding muscles. Hilum, calcifications and cystic change were categorized into absent or present. Vascularity pattern was classified as abnormal flow, hilar flow or absent flow. If peripheral or diffusely increased flow was observed, it was regarded as abnormal flow [6, 9].

FNA was performed by 1 of 3 radiologists with more than 5 years of experience in LN FNA (XFN, SYX, WZ), and the procedure was performed by freehand technique with 23G or 25G gauge needle and 5 ml syringe. Each LN was punctured for at least 3 times. Cytological smears were assessed by professional cytopathologists. LNs were included only if the FNA results were consistent with postoperative pathology.

### Statistical analysis

Mann–Whitney U test, χ2 and Fisher exact tests by univariate analysis were used to analyze clinical information, sonographic features on grey scale and color Doppler. In order to obtain the best cut-off points, receiver operating characteristic (ROC) curve analysis was used for long and short-axis diameter. A multiple logistic regression analysis by using binary logistic regression was applied to evaluate the malignancy risk for the independent features of sonographic features. We used the univariable analysis at a statistical significance level of *P* < 0.05 to determine of US features for the multivariate regression analysis. The B value, Wals χ, ratio (OR) and 95% CI were recorded. A *P* value < 0.05 was considered to indicate statistical significance. The area under the receiver operating characteristic curve (AUC) by logistic regression model was used to evaluate the predictive value of ultrasound in order to distinguish LNM-MTC and LNM-PTC. The statistical analysis was performed using SPSS version 25 (IBM Corporation, Armonk, NY, USA).

## Results

### Clinicopathological characteristics of LNM-MTC and LNM-PTC groups

The clinical characteristics of the patients with LNM-MTC and LNM-PTC were listed in Table 1. There were no significant differences in gender and location between LNM-MTC and LNM-PTC groups (*P* > 0.05). The mean age of patients with LNM-MTC was older than that of patients with LNM-PTC (46.81 ± 13.05 vs 39.09 ± 12.05, *P* < 0.001).

**Table 1:** Univariate analysis of clinical characteristics and ultrasound features of lymph nodes in the MTC and PTC groups

	MTC (n, %)	PTC (n, %)	*P* value
No. of LNs	52	234	
No. of patients	42	222	
**Clinical characteristics**			
Gender			
Male	17 (40.48)	60 (27.03)	0.079
Female	25 (59.52)	162 (72.97)	
Age	46.81 ± 13.05	39.09 ± 12.05	< 0.001 (Z = − 3.854)
Location			
Lateral	34 (65.38)	179 (76.50)	0.096
Central	18 (34.62)	55 (23.50)	
**Ultrasound features**			
Long-axis diameter	16.43 ± 10.36	12.32 ± 6.93	0.001
> 8.95	45 (86.54)	148 (60.91)	0.004^#^
< 8.95	7 (13.46)	86 (39.09)	
Short-axis diameter	8.53 ± 5.79	5.86 ± 2.61	< 0.001
> 7.85	24 (46.15)	43 (18.38)	< 0.001^#^
< 7.85	28 (53.85)	191 (81.62)	
L/S			
≤ 2	30 (57.69)	133 (56.84)	0.910
> 2	22 (42.31)	101 (43.16)	
Shape			
Irregular	3 (5.77)	5 (2.11)	0.150*
Regular	47 (94.23)	229 (97.89)	
Border			
Unsharp	2 (3.85)	4 (2.14)	0.300*
Sharp	50 (96.15)	230 (97.86)	
Hilum			
Absent	47 (90.38)	167 (71.37)	0.004^#^
Present	5 (9.62)	67 (28.63)	
Echogenicity			
Hyperechoic	19 (36.54)	67 (28.63)	0.261
Isoechoic or hypoechoic	33 (63.46)	167(71.37)	
Calcifications			
Present	11 (21.15)	139 (59.40)	< 0.001^#^
Absent	41 (78.85)	95 (40.60)	
Cystic change			
Present	2 (3.85)	29 (12.39)	0.085*
Absent	50 (96.15)	205 (87.61)	
Vascularity pattern			
Abnormal flow	31 (59.61)	101 (43.16)	0.031^#^
Hilar flow or absent flow	21 (40.38)	133 (56.84)	

*using Fisher's exact tests, others using chi-square (χ2); ^#^enrolled into Multivariate Logistic Regression

### Univariate analysis of US features in LNM-MTC and LNM-PTC groups

**Fig. 1:**
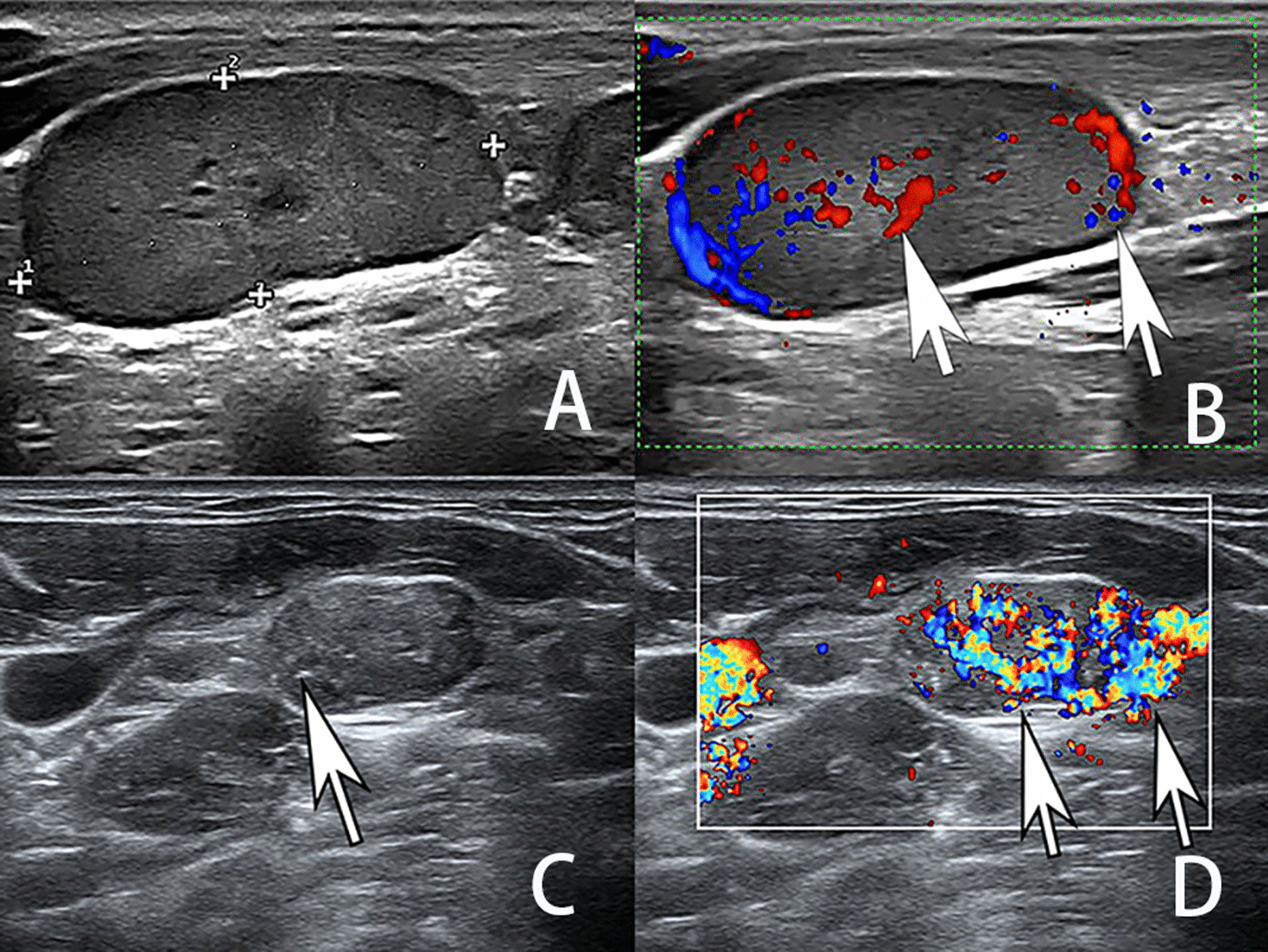
Ultrasonographic images of LNs. (**A**, **B**) A metastatic LN in a 53-year-old man with MTC. (**C**, **D**) A metastatic LN in a 29-year-old man with PTC. (**A**) The LN was located at left level IV, and the grey ultrasonographic images showed a LN measured 35.1 × 15.6 mm, with hyperechogenicity, L/S > 2 and absence of hilum. (**B**) Color Doppler showed that the LN had a mixed vascularity pattern (white arrow). (**C**) The LN was located at right level III. The grey ultrasonographic images showed a LN measured 15.7 × 9.0 mm, and it demonstrated absence of hilum, L/S ≤ 2, hyperechogenicity and microcalcifications (white arrow). (**D**) Color Doppler showed that the LN had a mix vascularity pattern (white arrow)

Table 1 showed the US features in LNM-MTC and LNM-PTC groups. LNM-MTCs were more likely to have the following US features (Table 1): L/S ≤ 2, regular shape, sharp border, isoechoic or hypoechoic, however, there were no significant differences in these features between LNM-MTC and LNM-PTC groups (*P* > 0.05). The ROC curve was used for the long and short-axis diameters. The cutoff points of long-axis diameter and short diameter were 8.95 and 7.85, and the areas under the curve were 0.641 and 0.664, respectively. Then, long and short-axis diameters were converted into binary variables. The mean sizes of LNM-MTC were larger than those of LNM-PTC (Fig. 1) in both long-axis diameter and short-axis diameter (*P* < 0.05). There were statistically significant differences in the features of hilum, calcifications and vascularity pattern between LNM-MTC and LNM-PTC (*P* < 0.05). By comparing the US characteristics of LNM-MTC in the lateral and central groups, it showed that the long axis diameter was significantly different between the two groups (*P* < 0.05), while the other characteristics had no differences (Table 2).

**Table 2:** Univariate analysis of ultrasound features of LNM-MTC in the lateral and central groups

	Lateral (n, %) (n = 34)	Central (n, %) (n = 18)	*P* value
Long-axis diameter			
> 8.95	32 (94.12)	13 (72.22)	0.041*
< 8.95	2 (5.88)	5 (27.78)	
Short-axis diameter			
> 7.85	16 (47.06)	8 (44.44)	0.857
< 7.85	18 (52.94)	10 (55.56)	
L/S			
≤ 2	17 (50)	13 (72.22)	0.123
> 2	17 (50)	5 (27.78)	
Shape			
Irregular	2 (5.88)	1 (5.6)	1.000*
Regular	32 (94.12)	17 (94.44)	
Border			
Unsharp	2 (5.88)	0 (0)	0.538*
Sharp	32 (94.12)	18 (100)	
Hilum			
Absent	31 (91.18)	16 (88.89)	1.000*
Present	3 (8.82)	2 (11.11)	
Echogenicity			
Hyperechoic	13 (38.24)	6 (33.33)	0.727
Isoechoic or hypoechoic	21 (61.76)	12 (66.67)	
Calcifications			
Present	7 (20.58)	4 (22.22)	1.000*
Absent	27 (79.41)	14 (77.78)	
Cystic change			
Present	2 (5.88)	0 (0)	0.538*
Absent	32 (55.88)	18 (100)	
Vascularity pattern			
Abnormal flow	22 (64.71)	9 (50)	0.304
Hilar flow or absent flow	12 (35.29)	9 (50)	

^*^using Fisher's exact tests, others using chi-square (χ2)

### Multivariate logistic regression analysis of US features in LNM-MTC and LNM-PTC groups

**Fig. 2:**
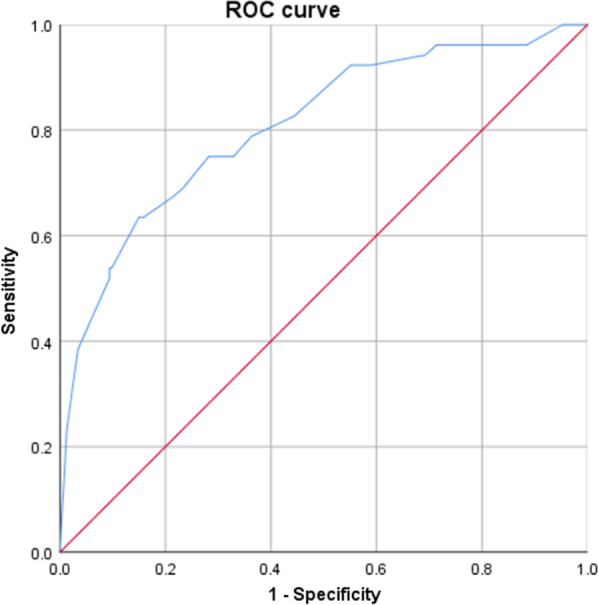
Receiver operating characteristic curve analysis of logistic regression model for predicting LNM-MTC and LNM-PTC. AUC was 0.808, and the 95% CI was 0.739–0.877

The results of multivariate logistic regression analysis were listed in Table 3. The ultrasonic indicators with statistical significance in the univariate analysis were included in the multivariate logistic regression analysis, and the malignancy risk was further evaluated for the independent features. Compared with LNM-PTC, long-axis diameter > 8.95 (OR = 3.543), short-axis diameter > 7.85 (OR = 2.708), absence of hilum (OR = 3.031) and absence of calcification (OR = 0.140) were more common in LNM-MTC (Fig. 1, *P* < 0.05), while there was no statistical significance in vascularity pattern (*P* > 0.05). AUC of discriminate ability between LNM-MTC and LNM-PTC was 0.808, with 95% confidence interval (CI) of 0.739–0.877 (Fig. 2).

**Table 3:** Odds ratios for the selected sonographic features by multivariate logistic regression analysis

	Multivariate analysis				
	B	Wals χ2	Odds ratio	95% CI	*P* value
Long-axis diameter	1.265	7.061	3. 543	1.394–9.007	0.008
Short-axis diameter	0.996	6.728	2.708	1.276–5.747	0.009
Hilum	1.109	4.457	3.031	1.083–8.487	0.035
Calcifications	-1.968	25.099	0.140	0.065–0.302	< 0.001
Vascularity pattern	0.528	2.227	1.696	0.847–3.395	0.136

## Discussion

As previously reported, MTC is a rare thyroid malignant tumor with an incidence of 0.11/100,000 in the population, which can be divided into sporadic and genetic types [10]. The C cells of MTC can secrete many kinds of substances, among which serum calcitonin is a valuable tumor marker of MTC. MTC is more aggressive than PTC, and it is prone to distant metastases including lung, liver, bone. Weber et al. reported that LNM-MTCs could be found in 75% patients who underwent total thyroidectomy and modified radical neck dissections [11]. Cervical lymph node dissection had certain complications, such as thoracic duct leak, injury to the recurrent laryngeal nerve, and hypoparathyroidism, and repeated operations were very traumatic to patients. Therefore, accurate preoperative diagnosis of LNM is of great significance for surgical plans.

In our study, there was no difference in gender between the two groups, however, patients with LNM-MTC were older than those with LNM-PTC. Similar results have also been reported in primary thyroid cancer. A previous study showed that patients with MTC were older than those with PTC [12]. No difference was observed in the location between LNM-MTC and LNM-PTC groups. However, more LNMs were located at the lateral region in both groups. LNMs of thyroid cancer usually spread to the central region first, followed by the lateral region [13], and central LNs dissection are routinely performed. Moreover, due to the poor US window and small size of LNs in the central region, the sensitivity of US in the detection of central LNMs was low [4]. Thus, many LNs in the central region might not be recognized, and FNA was not performed.

US is important for distinguishing benign and malignant LNs. The related ultrasonographic indicators of LN include size, L/S, shape, border, hilum, echogenicity, calcifications, cystic change and vascularization. LNMs of thyroid carcinoma are mainly manifested as microcalcifications, hyperechogenicity, cystic change and abnormal vascularization [6]. However, these features were commonly seen in LNM-PTC [14, 15], and they were rarely reported in LNM-MTC. To the best of our knowledge, the ultrasonic features of LNM-MTC have not been well documented in the literatures. In our study, no differences were observed in L/S, shape, border, echogenicity, cystic change and vascularity pattern between LNM-MTC and LNM-PTC groups. However, long-axis and short-axis diameter, hilum and calcifications were statistically different between these two groups. AUC showed good discrimination ability between LNM-MTC and LNM-PTC (AUC, 0.808; 95% CI 0.739–0.877). In our study, the mean long-axis and short-axis diameter in LNM-MTC were 16.43 ± 10.36 mm and 8.53 ± 5.79 mm, both larger than those of LNM-PTC. Compared with PTC, MTC had a higher degree of malignancy and invasion [12, 16], which might be the reason for larger sizes of LNs. Normal LNs usually have an echogenic hilum, while metastatic LNs usually present with absence of hilum [7, 14, 17]. The metastases of the tumor initially appear in the peripheral area of LN, but as the size of metastases become larger, the normal nodal tissue destroyed. When LNs were filled with tumor cells, hilum could not be recognized [18]. However, absence of hilum may be without significance in distinguishing LNM-PTC from normal LNs [6]. Due to its indolent nature, PTC usually develops slowly. When the medullary lymphatic sinuses have not been completely destroyed by tumor cells, hilum also exists in early LNMs [19]. The results in our study showed that absence of hilum was more commonly detected in LNM-MTC than in LNM-PTC. MTC is a moderate malignancy, and the tumor cells commonly grow with invasion. The difference might be due to the more aggressive biological behavior.

Nodal calcification plays an important role in differentiating benign and malignant LNs. It is also an important indicator of primary thyroid carcinoma [20]. Li et al. revealed that macrocalcification was more frequent in MTC, while microcalcification was more common in PTC [1]. Microcalcifications are usually round or concentric under the light microscope, with a tiny diameter of 10 to 100 μm, which correspond to the psammoma bodies on pathology. In MTC, macrocalcifications are associated with amyloid deposits [21]. Calcification in LNMs is generally rare, but it is common in LNM from thyroid cancer. Kim et al. found that there was no significant difference in calcifications between PTCs and MTCs [22]. To the best of our knowledge, calcification in metastatic LNs has not been compared between the two tumors. Our results showed that the incidence of calcifications in LNM-MTC group was significantly lower than that in LNM-PTC group (*P* < 0.05). In the LNM-PTC group, calcification was found in 59.40% (139/234) LNs, with a relatively high incidence. However, in LNM-MTC group, the incidence of calcifications was not high in LNM-MTC group, and it was detected in only 21.2% (11/52) cases. Due to the low prevalence, calcifications were not further subdivided into coarse and fine calcifications. The difference between the two groups indicated that although calcification was regarded as an ultrasonic feature of LNM-PTC, this sign may not be applied to LNM-MTC.

Hyperechogenicity and cystic change are also the characteristic manifestations of LNM-PTCs, and there were no differences between LNM-MTC and LNM-PTC in our study. The typical echogenicity of LNM from thyroid cancer is hyperechoic in the cortex compared to skeletal muscle [9]. This may be due to the deposition of thyroglobulin and clustering of the tumor cells in the LNs [9, 23]. LNM should also be suspected in patients with suspicious thyroid nodules when cystic change existed. The mechanism of cystic change in LNM may be related to necrosis [24]. Our results suggested that hyperechogenicity and cystic change can also be applied to LNM-MTC. Normal LNs usually had hilar flow, while metastatic LNs often showed peripheral flow or mixed flow, which was usually considered as abnormal flow [9]. Peripheral flow was a meaningful feature of LNM from thyroid cancer [25], which was caused by tumor angiogenesis or recruitment of capsular vessels [23]. In our study, abnormal flow was more frequent in LNM-MTC than in LNM-PTC, but the difference was not significant (*P* > 0.05).

It was reported that the ultrasonographic characteristics of LNM-PTC in the central and cervical groups were different [26]. The high frequency of malignant US characteristics in the lateral LNMs included partial cystic component, microcalcifications and diffusely increased echogenicity, however, these ultrasound findings may not apply to the central group. It was suggested that the taller-than-wide type was only malignant US feature in the central LNMs [26]. Previous studies also suggested that the sensitivity of US in central LNMs was lower than that of lateral cervical LNM in PTC, because the lymph nodes in the central region lacked characteristic sonographic appearances [4, 26]. The comparison of ultrasound images between the central and lateral LNs in LNM-MTC group was performed in our study, however, there were no differences in other indicators except the long-axis diameter. According to the results of our study, we suggested that the US features of LNM-MTC could also be applicable to the central LNMs.

There were several limitations in this study: First, due to the retrospective nature of this study, it is likely to have selection bias and restricted quality of images and data. Second, the sample size of LNM-MTC group was small, and a further study with a larger sample size should be performed.

## Conclusions

Although some ultrasonic features were overlapped in LNM-MTC and LNM-PTC, LNM-MTC tended to have the sonographic characteristics of larger size, absence of hilum, and less calcifications. When the primary tumor was MTC, LNs with these features should be suspected of metastatic lymph nodes.

## Data Availability

Due to patient privacy protection, materials and data are not publicly available, but are available from the corresponding author for reasonable request.

## Abbreviations

MTC: Medullary thyroid carcinoma
LNM: Lymph node metastasis
US: Ultrasound
LN: Lymph node
LNM-PTC: Lymph node metastases from papillary thyroid cancer
LNM-MTC: Lymph node metastases from medullary thyroid cancer
L: Long-axis diameter
S: Short-axis diameter
L/S: Long-axis diameter/short-axis diameter
ROC: Receiver operating characteristic
OR: Odd ratio
CI: Confidence interval
